# Peritoneal Surface Malignancies Originating From Urachal Carcinoma: Case Reports and Review of the Literature

**DOI:** 10.1007/s13193-022-01679-4

**Published:** 2022-12-17

**Authors:** Paulien Van Breusegem, Geert Verswijvel, Sabine Fransis, Kurt Van der Speeten

**Affiliations:** 1 Department of Surgical Oncology, Hospital Oost-Limburg, Schiepse Bos 6, 3600 Genk, Belgium; 2 Department of Radiology, Hospital Oost-Limburg, Genk, Belgium; 3 Department of Pathology, Hospital Oost-Limburg, Genk, Belgium; 4 Faculty of Medicine and Life Sciences, BIOMED Research Institute, University Hasselt, Hasselt, Belgium

**Keywords:** Urachal carcinoma, Peritoneal carcinomatosis, Cytoreductive surgery, HIPEC

## Abstract

Urachal carcinoma (UC) is a rare and aggressive tumor arising from the urachal remnants, with the potential for peritoneal dissemination. Patients diagnosed with UC often have a poor prognosis. To date, there is no standardized treatment. Our objective is to present two cases of patients with peritoneal carcinomatosis (PC) secondary to an UC, who were treated with cytoreductive surgery (CRS) and hyperthermic peroperative intraperitoneal chemotherapy (HIPEC). A review of the literature on CRS and HIPEC in UC suggests CRS and HIPEC to be a safe and viable treatment option. Two patients with PC of UC underwent CRS and HIPEC in our institution. All available data were gathered and reported on. A literary search was carried out to find all available cases of patients with PC secondary to UC treated with CRS and HIPEC. Both patients underwent CRS and HIPEC and are currently free of recurrence. Literature research revealed nine other publications adding up to a total of 68 additional cases. CRS and HIPEC can provide satisfactory long-term oncological outcome with acceptable morbidity and mortality rates in patients with PC of urachal origin. It should be considered as a safe and feasible treatment option with curative potential.

## Introduction

Urachal carcinoma (UC) is a rare and aggressive cancer that often presents in advanced stages of disease. It accounts for only 0.35–0.70% of all bladder cancers and 0.01% of all adult malignancies and has an incidence of approximately one case per million per year [1]–[5]. UC can present at any age. A review with meta-analysis of 1010 cases by Szarvas et al. [1] showed a median age at presentation of 52 years (range 20–90 years). In addition, there are a few cases where UC is described in newborns and children. The extravesical and extraperitoneal locations of UC make for a clinically silent character, resulting in diagnosis often at advanced tumor stages. The meta-analysis by Szarvas et al. reported lymph node metastasis in 17% and primary metastatic disease in 21% of patients [1, 6]. Most often distant organ metastases present in the lungs, the bones, and the peritoneum [1, 6].

The aim of this manuscript is to present two cases of patients with an UC with isolated peritoneal metastases. Both patients were treated with cytoreductive surgery (CRS) and hyperthermic peroperative intraperitoneal chemotherapy (HIPEC). Second, a review of the available medical literature on the treatment of this entity is presented.

## Case Presentation 1

A 49-year-old man was referred to our institution because of findings of a large, mostly preperitoneal located, mass. In his surgical history, we only withheld vasectomy. Ultrasound and computed tomography (CT) scan showed a large (approximately 8 cm) circumscript mass lesion in the hypogastrium. The base of the tumor originated from the bladder dome, not delineable from the detrusor muscular layer. Tiny reticular septations crossed the lesion, and a few punctate calcifications were demonstrated in the center and the periphery of the tumor. Peritoneal carcinomatosis (PC) could not be excluded. A need for surgical removal of the lesion with debulking and HIPEC could be concluded.

A median xiphopubic skin incision with excision of the umbilicus was performed. A limited amount of gelatinous mass was found immediately upon opening the abdomen supraumbilically. A sample was taken for cytology. Further exploration of the abdomen revealed a large mass extending from the umbilicus into the bladder with marked transserosal growth and breakthrough. The final peritoneal carcinomatosis index (PCI), according to Sugarbaker [7] was 7. The entire mass was resected en bloc, consisting of the peritoneum of the lower hemi-abdomen, the umbilicus, anterolateral parietal peritonectomy, partial cystectomy, and rectal resection. The bladder trigonum could be preserved. The bladder was left open for exposure to the HIPEC since a massive amount of mucus was found in it. An omentectomy and cholecystectomy were also performed. CC0-CC1 resection was achieved.

HIPEC was performed with an open technique for 90 min. The abdomen was filled with a peritoneal dialysis fluid at 41.5 °C. Bidirectional (combined intraperitoneal and intravenous) chemotherapy was initiated: 5-fluorouracil (5FU) at a dose of 400 mg/m^2^ (total dose of 788 mg) in 250 ml NaCl, and leukovorin at a dose of 20 mg/m^2^ (total dose of 39.4 mg) in 250 ml NaCl 0.9% was administered as a fast bolus through two separate IV lines. Mitomycin of 15 mg/m^2^ in combination with doxorubicin of 15 mg/m^2^ was added to the HIPEC circuit for a total dose of 29.6 mg each. Body temperature rose from 35.8 to 39.4 °C during the HIPEC. At the end, all perfundates were aspirated. After meticulous hemostasis, the bladder was closed; a nutritional jejunostomy was placed; and the abdomen was closed on drains.

Postoperative on day one, an Adams-stokes syndrome with three episodes of total AV block without escape rhythm and pauses of up to 20–25 s was observed. No clinical features and no signs of heart decompensation were noted. A transthoracic ultrasound showed normal systolic ventricular function without significant valvular disease. A temporal pacemaker was placed. During the further CCU admission, however, no recurrent AV block was observed, and therefore there was no need for definitive pacing.

Discharge from the hospital followed on day 11. At present (October 2022), 12 years after CRS and HIPEC, the patient is alive with no evidence of disease. The patient did not receive any adjuvant therapy.

**Fig. 1:**
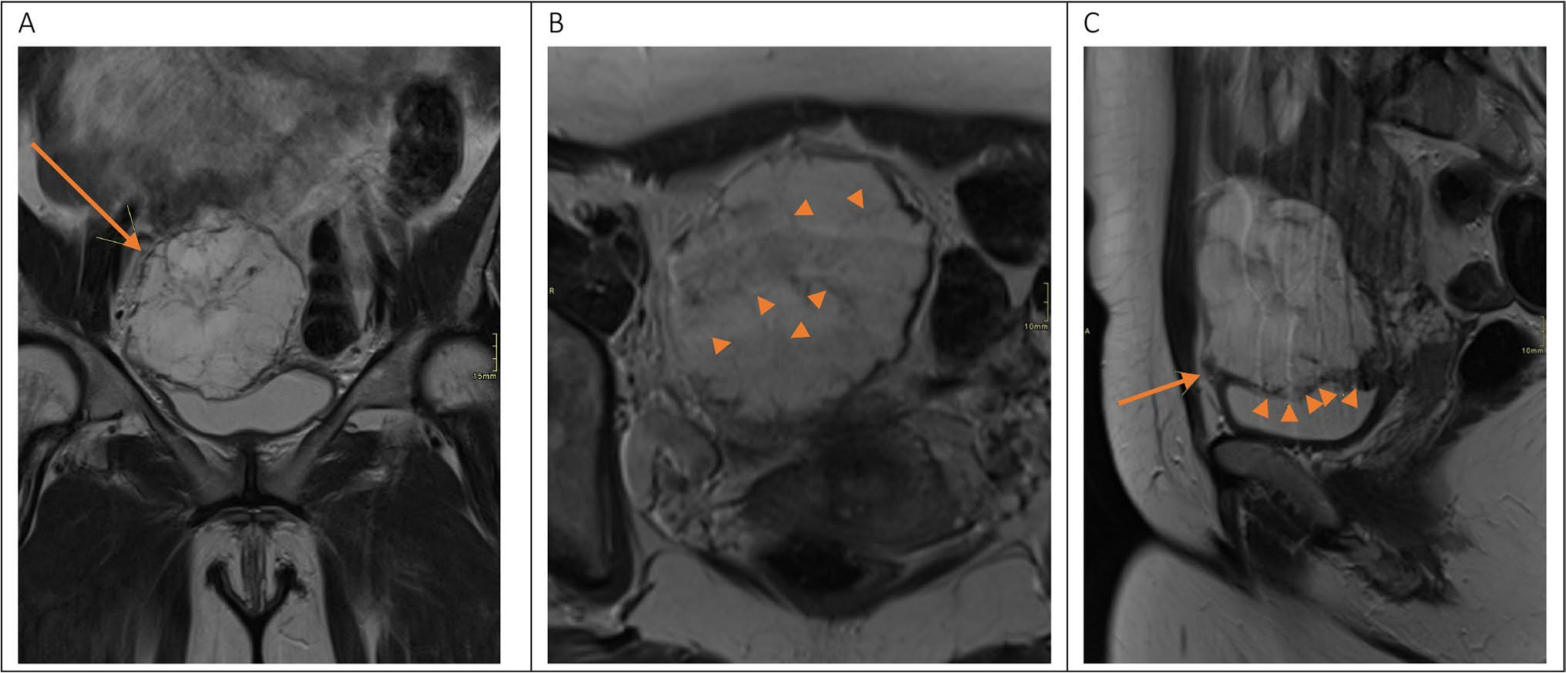
**A** T2WI in the coronal plane demonstrating a large (8 cm) mass lesion (arrow) in the pelvis, not to be delineated from the bladder dome. **B** T2WI in the axial plane. The border of the lesion is slightly irregular and tiny reticular structures seem to cross the tumor (short arrows). **C** T2WI in the sagittal plane demonstrates that the tumor has a broad-based implantation in the dome of the urinary bladder with interruption of the muscle layer (short arrows). Furthermore, the origin of the medial umbilical ligament (arrow) cannot be delineated from the tumor

Pathology (Fig. 1) revealed a mucinous adenocarcinoma (colloid subtype, with the bladder an intestinal subtype) measuring 12 cm × 5.5 cm × 6.5 cm and opening into the bladder lumen. The presence of gelatinous material bordered by a pseudostratified intestinal type epithelium with hyperchromatic nuclei and increased mitotic activity could also be found in the peritoneum of the right lower quadrant. The findings are fitting for an UC. However, morphologically and immunohistochemically, a peritoneal metastasis of gastrointestinal origin could not be excluded.

## Case Presentation 2

A 42-year-old woman was referred with irritative voiding symptoms, mostly frequency and urgency. Imaging revealed an abdominal lesion. In the surgical history we noticed gastric banding and tubal ligation.

**Fig. 2:**
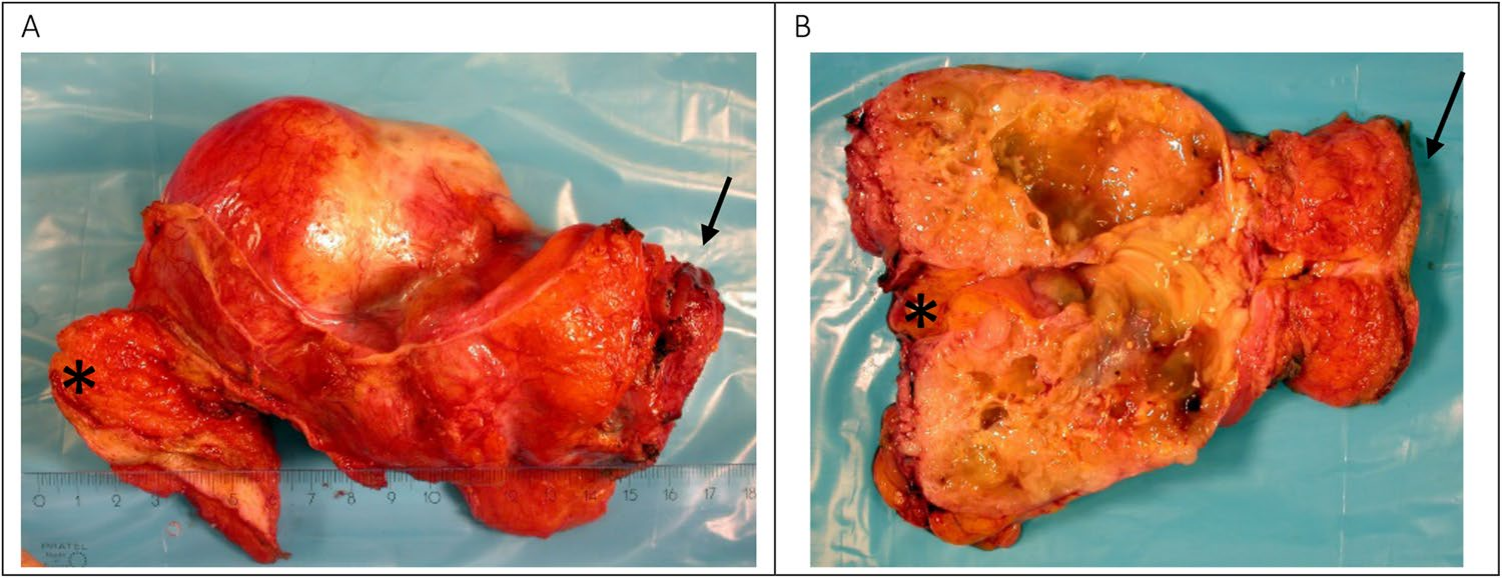
Intra-operative imaging of the excised specimen showing partial cystectomy containing urachal adenocarcinoma involving the bladder dome (arrow) and umbilical ligament (*)

The ultrasound of the pelvic cavity demonstrated a voluminous mass cranial to the bladder with an inhomogeneous pattern and the impression of calcifications. Additionally, a magnetic resonance imaging (MRI) (Fig. 2) of the abdomen was performed revealing a mass measuring 7.5 cm × 6.5 cm × 8 cm, developing on or arising from the dome of the urinary bladder. Invasion of the uterus, the peritoneum, and the adjacent small intestine could not be excluded. The morphology and signaling features on MRI were suggestive of an underlying tumor of mucinous origin. In retrospect, a CT scan performed 5 years prior, revealed a small, nodular lesion with a diameter of 10 mm on the bladder dome, suspect of a calcified urachus remnant. Lung metastases were excluded on thoracic CT.

Based on the MDT discussion; the patient was counseled for surgical exploration, CRS, and HIPEC in case of PC.

**Fig. 3:**
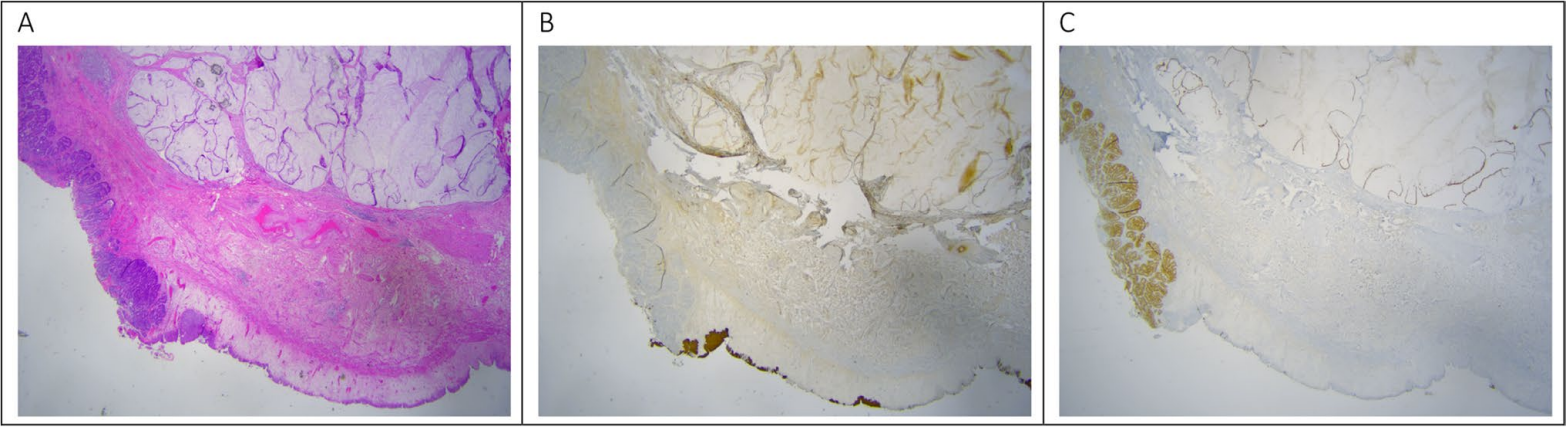
Intestinal type of adenocarcinoma in the bladder wall. **A**. Hematoxylin and eosin (HE) stain, 1.25 × magnified. An intestinal type adenocarcinoma with mucinous differentiation at the top consisting of mucus containing strips of atypical intestinal type epithelium is present at the left bottom. At the right bottom multi-layered urothelium without atypia is seen. **B**. CK7 immunohistochemical staining, 1.25 × magnified. The tumor cells (both the classical intestinal component and the mucinous component) do not stain, whereas the normal urothelium does. **C**. CDX 2 antibody staining, 1.25 magnified. Nuclear enhancement of the adenocarcinoma (both the classical type and the mucinous component) for CDX 2; the urothelium does not stain

A median xyfopubic incision was performed. Following adhesiolysis around the tubing of the gastric banding, the abdomen was explored. The Prior surgical score [8] was 0. The PCI, according to Sugarbaker [7], was 5 (zone 0 = 1, zone 5 = 3, zone 6 = 1). A large tumoral mass, originating from the bladder extending to the umbilicus could be visualized: consistent with UC. T4-transserosal progression with adjacent satellite lesions on the left peritoneum and more cranially was detected. A significant amount of ascites was present. Samples were sent for cytology. Anteroparietal peritonectomy was carried out towards the bladder. The connection of the UC to the bladder cavity was identified. The bladder and the dome of the bladder were opened and excised. The fistulization to the bladder was demonstrated and completely excised. Peritonectomy of the bladder posteriorly and further dissection distally was executed. The specimen (Fig. 3) was sent for pathological examination. Subsequently, a total omentectomy and appendectomy were performed. A CC-0 cytoreduction was achieved.

The bladder was left open after clamping the urinary catheter to flush the bladder later during the HIPEC. HIPEC was performed with the open technique for 30 min. The abdomen was filled with a saline solution at 42.5 °C. Bidirectional (combined intraperitoneal and intravenous) chemotherapy was initiated: 5FU at a dose of 400 mg/m^2^ (total dose of 656 mg) in 250 ml NaCl was administered via the central line as a bolus. Leukovorin at a dose of 20 mg/m^2^ (total dose of 32.8 mg) in 250 ml NaCl of 0.9% was administered as a bolus via the peripheral line. Oxaliplatin of 460 mg/m^2^ was added to the HIPEC circuit for a total dose of 755 mg. In this case, doxorubicin was not used again, as the patient in case 1 had a total AV block, presumably as a side effect of the doxorubicin. In the literature, at the time of occurrence, a total AV block was described as a side effect of doxorubicin when used as systemic therapy due to accumulation. [9] However, this was never described in HIPEC. After this case, a switch was made to Oxaliplatin as chemotherapy for HIPEC. Since there is no standardized therapy for the treatment of UC, the decision to use Oxaliplatin was based on the literature at the time and consultation with colleagues.

At the end of the HIPEC, all perfundates were aspirated. After meticulous hemostasis, the bladder was closed; a nutritional jejunostomy was placed; and the abdomen was closed on drains. The total duration of the operation was 5 h and 10 min.

There was a 3-day ICU stay with initial nausea. On day 5, the oral intake could be gradually resumed. Following a cystogram on day 7; the transurethral catheter was removed. Uneventful discharge followed on day 9. Currently (October 2022), 5 years after CRS and HIPEC, the patient is alive with no signs of disease recurrence. The patient did not receive any adjuvant therapy.

**Fig. 4:**
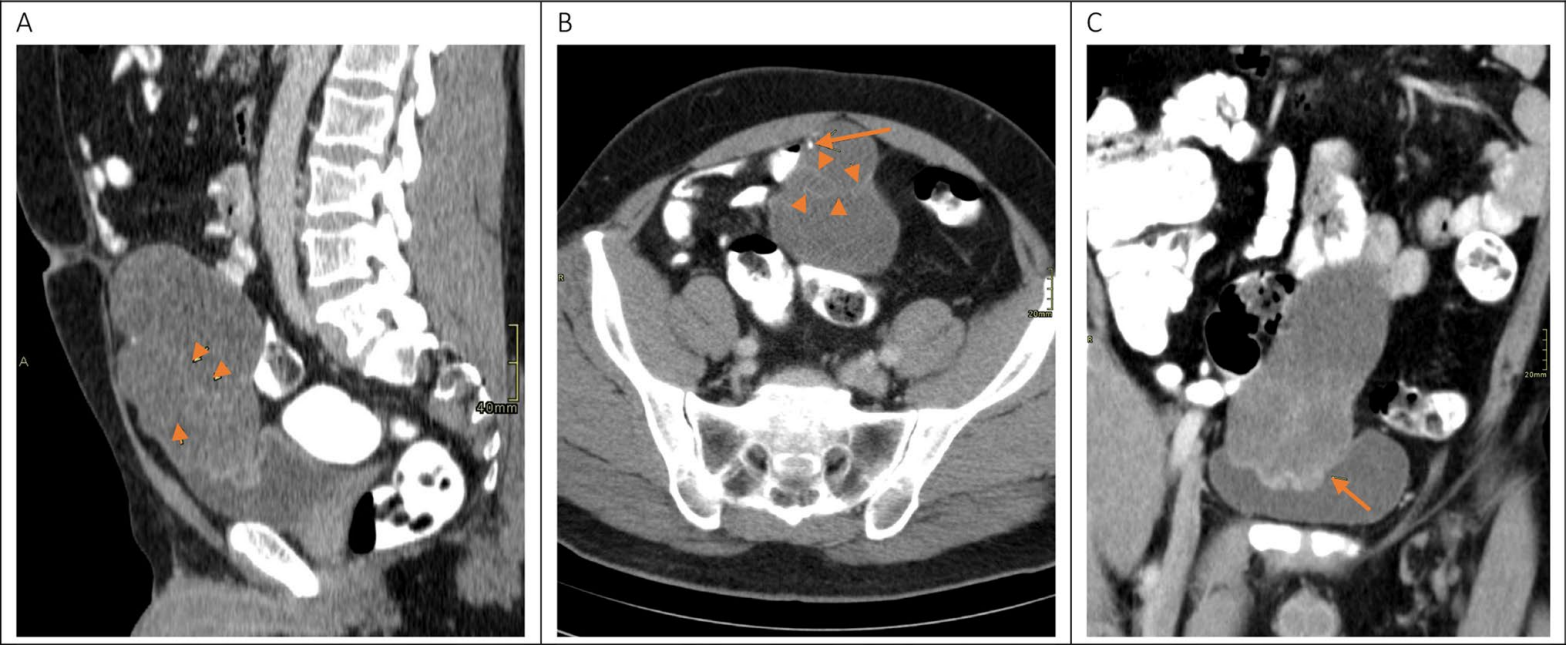
**A** CT reconstruction in the sagittal plane. A large hypodense lesion is demonstrated in the hypogastrium. This tumor arises from the anterior bladder dome to the level of the umbilicus. The middle umbilical ligament cannot be delineated from the lesion. Some tiny reticular septations are seen in the lesion (arrows). **B** CT reconstruction in the axial plane. A punctate calcification (arrow) is demonstrated in the periphery of the tumor. Hair thin contrast enhancing septations are crossing the tumor mass. **C** CT reconstruction in the coronal plane. The base of the tumor cannot be delineated from the muscular layer of the urinary bladder (arrow)

Pathology (Fig. 4) revealed a mixed mucinous and intestinal adenocarcinoma (7.5 cm) present in bladder mucosa, lamina propria, muscularis propria of the bladder, and in the perivesical fat. The differential diagnosis considered a primary bladder adenocarcinoma, a metastasis of an adenocarcinoma of intestinal origin or an UC. Since no remnant of the urachus could be found by the pathologist, it was not possible to distinguish based on histology and immunohistochemistry. The diagnosis had to be made clinically and radiologically. Pathological staging demonstrated a stage IIIa, according to Sheldon et al. [4] for UC.

## Background

The urachus is a tubular remnant of embryonic development, derived from the involution of the allantois and the cloaca [2]. After its involution in the third trimester of pregnancy, the urachus remains as the medial umbilical ligament and has no longer a physiological function [2, 10].

Autopsy studies suggest that the urachus canal fails to completely obliterate in one-third of adults. Partial persistence may lead to various anomalies including malignant transformations [1, 10]. The urachus consists of three distinguishable layers: an epithelial canal lined by urothelium, submucosal connective tissues, and an outer layer of smooth muscle. From each of these layers a neoplasm can arise. [11].

## Clinical Parameters and Imaging

**Fig. 5:**
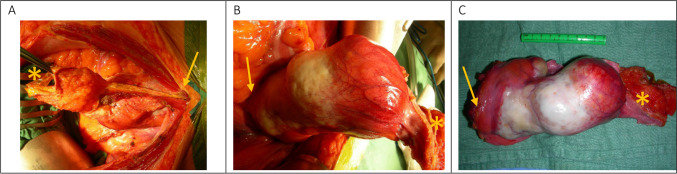
Intra-operative imaging of the excised specimen showing partial cystectomy containing urachal adenocarcinoma involving the bladder dome (arrow) and umbilical ligament (*)

**Fig. 6:**
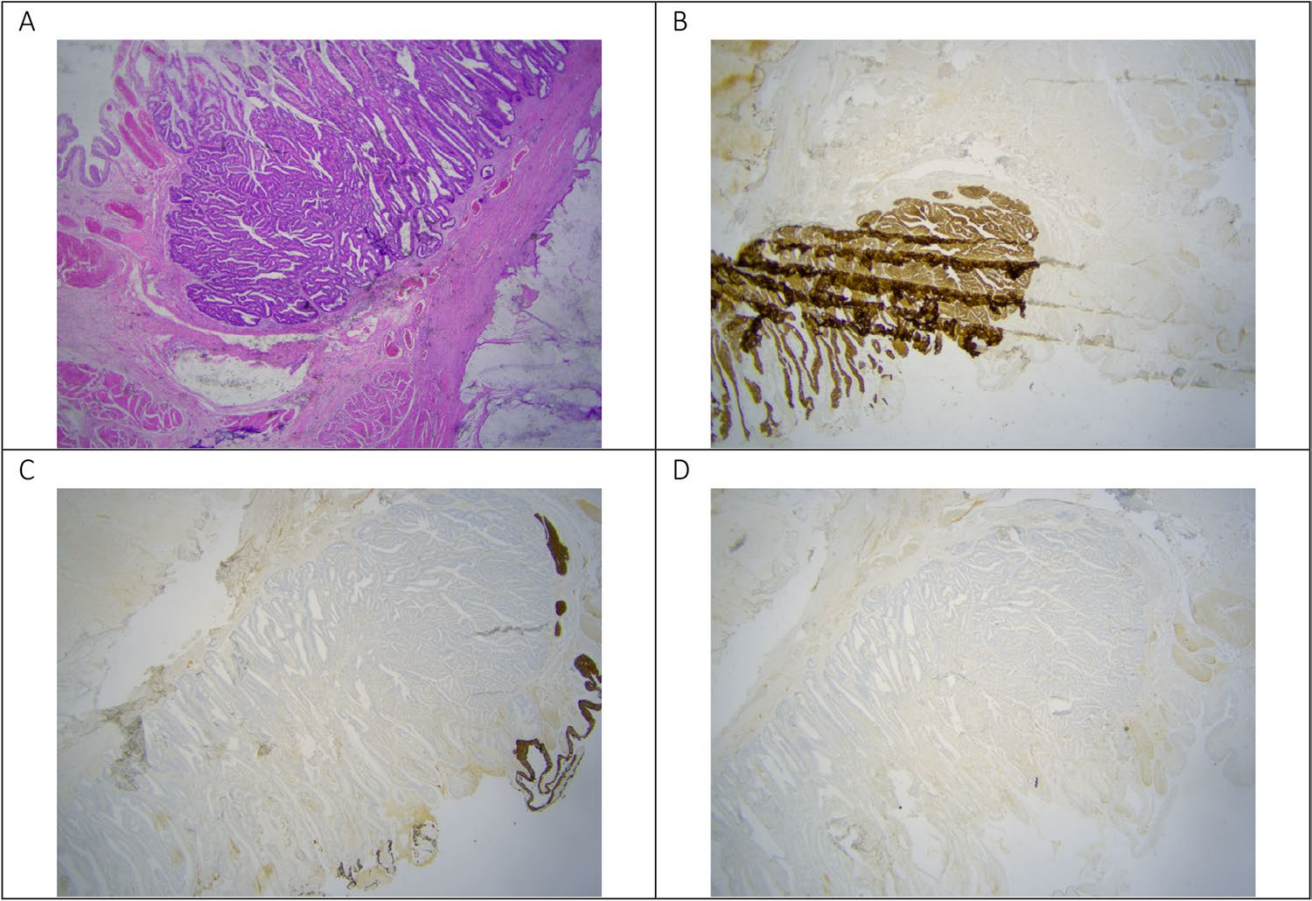
**A** HE staining. Urothelium on the left edge, interrupted by an intestinal type of adenocarcinoma with mucinous differentiation to the right. **B** Antibody CK20 staining, 1.25 × magnified. The intestinal type adenocarcinoma stains with this antibody, both the classical component and the mucinous component. **C** Antibody CK7 staining, 1.25 × magnified. Only the covering urothelium stains. **D**. CDX2 staining, 1.25 × magnified. The tumor cells do not express CDX2

Criteria for diagnosis of UC were first described by Sheldon et al. [4] in 1984 and later on modified by Gopalan et al. [11]. These include the following characteristics: 1. The tumor is located in the anterior wall/dome of the bladder, 2. Epicenter is located in the bladder wall, 3. Absence of widespread cystitis cystica/glandularis beyond the anterior wall/dome, and 4. Lack of a known primary tumor elsewhere [4, 11] (Figs. 5 and 6).

Patients most often present with vague and non-specific symptoms. The most common sign of UC is macroscopic or microscopic hematuria, followed by a palpable suprapubic mass, abdominal pain, dysuria, and mucosuria. Less common symptoms include irritative voiding symptoms, umbilical discharge (e.g., blood, pus, or urine), vaginal discharge, and non-specific symptoms (nausea, vomiting, anorexia, fever, or weight loss) [1, 3, 4].

Besides, medical history and physical examination cystoscopy is a very important diagnostic tool for UC. In addition to confirming that a tumor is present, a cystoscopy can also specify the location of the tumor in the anterior wall or bladder dome. At early stages the tumor is often covered with normal mucosa. Pressing the suprapubic area can cause mucus eruption and uncover the hidden tumor. Biopsy for anatomical pathological examination can be taken [1, 12] (Fig. 1).

Imaging has a very important role in the further workup and staging of UC [10]. Ultrasonography often reveals a supravesical, irregular. and inhomogeneous mass in the midline. Mucin-producing UCs, like other mucinous adenocarcinomas, can contain calcifications that can be detected by ultrasound, as well as by a CT-san or MRI. Calcifications at the bladder dome/midline are therefore considered pathognomic for UC [1, 3, 13, 14].

CT and MRI can be very useful in TNM staging and diagnosing urachal tumors. They might provide information about the local extension and lymph node invasion or distant metastasis. However, CT can only detect half of the bladder wall invasions, demonstrating its limited value in estimating tumor invasion. The combination of positron emission tomography (PET) with CT provides more accurate information on staging [1, 3, 12, 14, 15]. Furthermore, Stokkel et al. describes diagnostic laparoscopy including abdominal cytology as an important tool in staging UC. The presence and extent of PC are import parameters when considering therapeutical options [16].

Tumor markers such as CA 125, CA 19.9, and CEA can be measured for diagnostic or disease monitoring purpose. They can be elevated due to PC, a frequent finding in metastatic disease [1, 6, 17].

## Clinical Staging

Several systems have been proposed for staging of UC. An overview of all staging systems can be found in Table 1. In 1984, Sheldon et al. [4] first proposed a staging system based on the localization of the tumor. The system consists of eight categories and classifies urachal cancer confined to the urachal mucosa as early stage, whereas late-stage urachal cancer extends to extraurachal structures. It is still the most commonly used system. [5, 18] The Mayo clinic has put forward a second staging system. The initial proposal was made in 1993 by Henly et al. [19] but was subsequently modified by Ashley et al. [20] in 2006. From then on, it was referred to as the Mayo staging system. Both the Sheldon as the Mayo staging system have proven to provide a good prediction of survival. However, due to the high number of categories in the Sheldon system, it was found to be over-specified and too complex. With only half the number of categories, the Mayo staging system provides a more balanced distribution of UC patients between stages and therefor outperformed the Sheldon staging system with a more applicable risk-stratification [1, 19, 20].

**Table 1:** Staging systems for UC

Sheldon staging (Sheldon et al. [4]) Stage I Stage II Stage IIIA Stage IIIB Stage IIIC Stage IIID Stage IVA Stage IVB	Urachal cancer confined to urachal mucosa (no invasion beyond urachal mucosa) Urachal cancer with invasion confined to urachus itself Local urachal cancer extension to bladder Local urachal cancer extension to abdominal wall Local urachal cancer extension to peritoneum Local urachal cancer extension to viscera other than bladder Metastasis to regional lymph node Metastatic urachal cancer to distant sites
Mayo staging (Szarvas et al. [1]) Stage I Stage II Stage III Stage IV	Tumor confined to urachus and/or bladder Tumor extending beyond the muscular layer of urachus and/or the bladder Tumor infiltrating the regional lymph node Tumor infiltrating non-regional lymph nodes or other distant sites
Ontario staging (Bao et al. [18]) T1 T2 T3 T4	Urachal cancer confined to urachus Urachal cancer confined to bladder Urachal cancer invading surrounding fat Urachal cancer extending to the peritoneum
TNM staging (Shao et al. [3]) Tis T1 T2 pT2a pT2b T3 N M	A tumor localized to the urachal mucosa and no invasion to the basal membrane (carcinoma in situ) A tumor with invasion through the basal membrane Tumor invades into muscle of the bladder Tumor invades deep muscle of the bladder (outer half) Tumor invades the superficial muscle of the bladder (inner half) Tumor invades perivesical fat, abdominal wall muscle (in cases of extravesical urachal cancers) Followed the traditional TNM staging system Peritoneal implants were considered metastasis

Less well-known is the Ontario staging system, another simplified classification of UC involving four stages. Infiltration of regional lymph node and distant metastases are considered separately. With the idea that T1-T3 tumors are potentially curable with the appropriate surgery, and T4 tumors, on the other hand, are unlikely to be cured even with very aggressive surgery, Herr et al. [21] came up with the idea for a dichotomous staging. The staging of primary UCs can simply be described as restricted to the urachus, bladder, and perivesical tissue versus intraperitoneal spread of disease [3, 18, 21].

The Tumor Node Metastasis (TNM) system is also used by several authors because of its consistency and universal application. Shao et al. [3] proved the TNM system to be a good predictor for survival as well [3, 22].

## Pathology

Normal urachus in adults is primarily lined by transitional epithelium; however, UC is most often histologically characterized as adenocarcinoma (80–90%). A possible theory for this occurrence states that the enteric epithelial rest within the urachal remnant left behind from the cloaca underwent malignant transformation. Another hypothesis suggests columnar metaplasia of the urachal mucosa [2].

Table 2 provides an overview of the variety of epithelial lesions of the urachus. Within the group of glandular neoplasms there is an important difference between cystic and non-cystic adenocarcinomas. The latter group represents 83% of all UC. The most commonly encountered subtype being the mucinous type is followed by the enteric type. Other, more rare, subtypes include signet ring cell type, the not otherwise specified (NOS) type, and the mixed patterns [1, 2, 5, 23].

**Table 2:** Epithelial urachal neoplasms (adapted from Reis et al. [5] and Paner et al. [23])

	Subtype	Clarification	Occurrence
Glandular neoplasms			
Villous adenoma			
Non-cystic adenocarcinomas (83%)	Mucinous (colloidal)	Abundance of extracellular mucin with at least focal clusters of tumor cells floating within	57%
	Enteric (intestinal)	Dominant cribriform and/or glandular pattern with pseudostratified epithelia	15%
	Not otherwise specified (NOS)	General adenocarcinoma patterns are present without clear mucinous or intestinal morphology	14%
	Mixed	Neither the mucinous nor the intestinal pattern is dominant	8%
	Signet ring cell	Signet ring cells are the leading component	6%
Cystic adenocarcinomas	Mucinous cystadenoma	Lined by a single layer of mucinous columnar epithelium devoid of atypia	13%
	Mucinous cystic tumor of low malignant potential (MCTLMP)	With areas of epithelial proliferation, including papillary formation and low-grade atypia	65%
	MCTLMP with intraepithelial carcinoma	Significant epithelial stratification and unequivocal malignant cytological features, often with stroma-poor papillae and cribriform pattern	6%
	Mucinous cystadenocarcinoma: *with microinvasion*	Stromal invasion < 2 mm and comprising < 5% of the tumor	13%
	*Frankly invasive*	More extensive invasion	3%
Non-glandular neoplasms			Coexisting component in 4–8%
Urothelial neoplasms			
Squamous cell neoplasms			
Neuroendocrine neoplasms			
Mixed carcinomas			

The prognostic significance of the different subtypes remains unclear. Some publications show the signet ring cell type to demonstrate worse prognostic characteristics [1, 5]. The cystic adenocarcinomas, on the other hand, exhibit a more favorable prognosis with better progression-free survival than non-cystic adenocarcinomas. They are often detected as a tumorous mass and carry the risk of developing a Pseudomyxoma peritonei [5, 23].

It is important to note that a diagnosis of UC should never be made based on histopathology alone. It appears that the enteric type is histologically very similar to colorectal adenocarcinomas. In addition, the mucinous type is histologically indistinguishable from ovarian, pancreaticobiliary, and appendix carcinomas. Both types also occur in invasive urothelial carcinomas. Signet ring cell type also corresponds to the histomorphology of signet ring cell carcinomas of stomach, ovarian, and other sites [5, 23]. Immunohistochemical staining of UC demonstrates significant overlap with a primary bladder adenocarcinoma as well as with metastatic colorectal adenocarcinoma. CK20 and CDX2 are almost always expressed in UC, and in a more variable extend CK7, β-catenin and high molecular cytokeratin are expressed as well [11].

It can therefore be concluded that diagnosis should always be correlated with clinical presentation and medical imaging. In addition, the possibility of metastatic disease originating from other sites must also always be considered [5, 23].

## Therapy

The optimal treatment is still a matter of debate and should consider the staging of the tumor, the general condition of the patient and the wishes of the patient.

The mainstay of therapy for a localized and surgically resectable UC is surgery. A margin-negative, en bloc resection with complete removal of the urachus remnant and the umbilicus has a significant impact on survival and is described as a recurrence-risk reducing procedure, according to Szarvas et al. [1]. Whether it is best to perform a partial or a radical cystectomy is still up for discussion. Both provide similar oncological results, but with the organ-sparing partial cystectomy a higher quality of life is seen for the patient. The latter is therefore preferred [1]–[3] of the radical or partial cystectomy, it is advised by Szarvas et al. [1]. Considering the fact that lymph node positivity (without distant metastasis) shows a similar negative effect on survival as the presence of distant metastasis, resection makes sense as part of the evaluation of staging and prognosis. It may be even therapeutic for at least some patients [1, 3, 20].

Regarding the technique, standard use is made of the open technique. However, cases are described in which the patient is treated through the laparoscopic or even robot-assisted technique. These are described as safe, feasible, and minimally invasive with fewer morbidities in terms of postoperative pain and cosmesis [3, 24].

In patients with metastatic disease, the best option of potential survival prolonging therapy is systemic treatment. A separate entity in this is the patients with solitary PC. In these patients, CRS in combination with HIPEC has been described as a successful disease-free survival prolonging treatment option in several cases. It may even be offered as a potentially curative option for this specific group of patients [25]–[33].

**Fig. 7:**
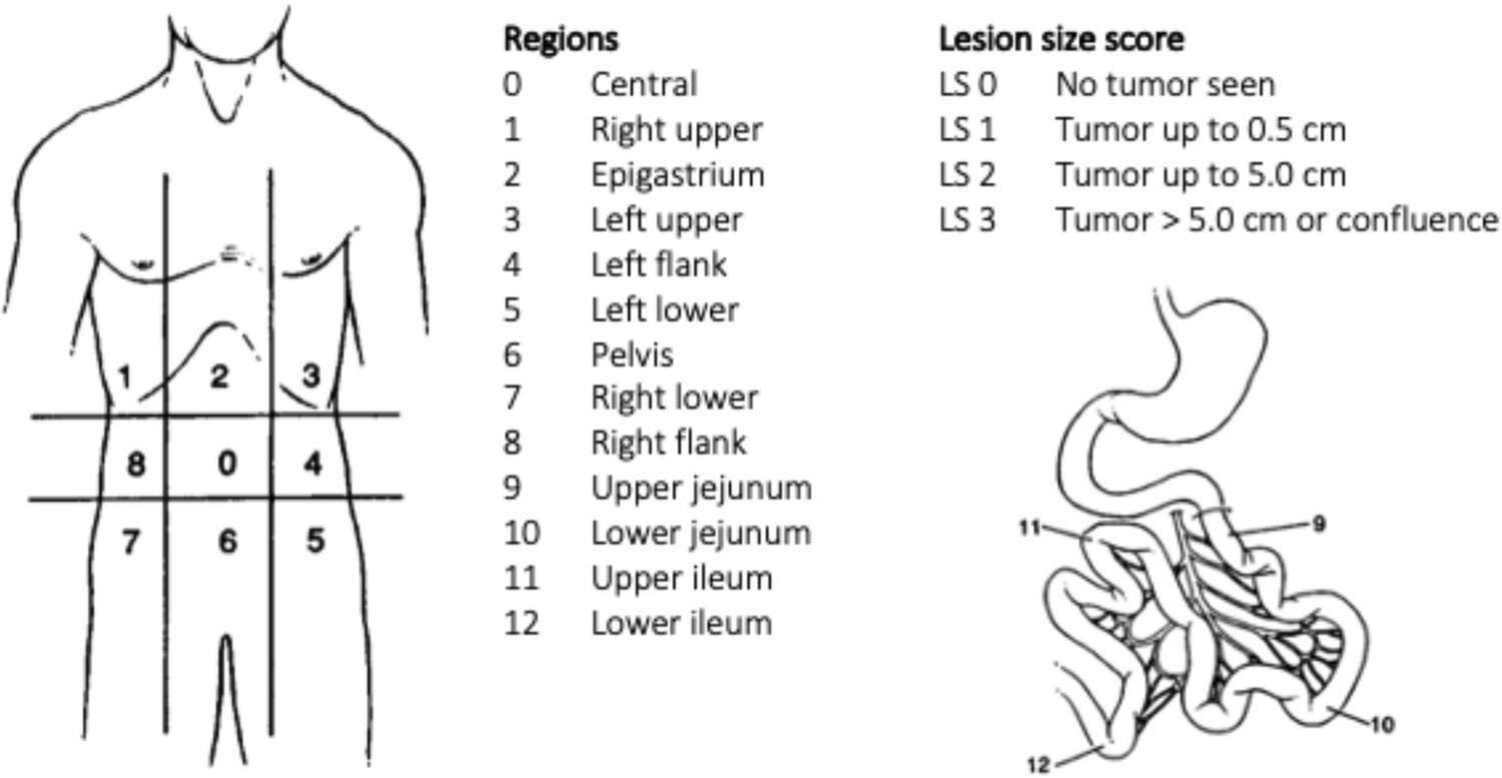
Peritoneal carcinomatosis index (PCI) scoring system, adapted from Sugarbaker et Al. [7]

CRS is started by systematically inspecting the abdominal cavity. An assessment is made of the extent of peritoneal disease using the PCI (Fig. 7). This diagnostic and prognostic tool gives a score from 0 to 39, based on the distribution and size of cancerous lesions. The abdomen is divided into thirteen regions, each receiving a score of 0–3 based on the largest tumor size in that region. The higher the score, the more extensive the disease is [7]. The aim of CRS is to obtain a complete resection of all macroscopic tumor lesions, achieved through peritonectomy procedures and visceral resections. The completeness of resection is scored by the CC-score. CC0 indicates no visible tumor remnants; CC1 indicates tumor nodules persisting < 0.25 cm; CC2 indicates tumor nodules between 0.25 and 2.5 cm, and CC3 indicates tumor nodules > 2.5 cm [7, 25]–[27]. After maximal cytoreduction, HIPEC is started. Chemotherapy is introduced via perfusion circuit in the abdominal cavity at a temperature of above 40 °C. The duration of the procedure and the type of chemotherapy differ between different studies [25]–[27, 34]. Because of the rarity of this disease there is not yet a standardized procedure described.

### Radiotherapy

Due to the limited radiosensitivity of UCs, radiotherapy has only a very limited place in its management. In a study conducted by Mertens et al. [25], radiotherapy is used as a local treatment prior to surgical resection. Neoadjuvant, 20 × 2 Gy of external radiation was administered. In addition, intra-operative use of brachytherapy was made on the suture line of the bladder. This was done because of the importance of the surgical margin status. This treatment, however, is not recommended by any universal guideline [25].

As with other tumors, metastases to the bone are possible. In a palliative setting, radiotherapy has been successfully used as an antalgic treatment [2, 25]. However, there are some studies that describe radiotherapy as an integral part of the treatment. For example, Sheldon et al. [4] described in 1984 radiotherapy as an adjuvant therapy to be effective in a few patients with advanced lesions. In particular, an extension of life is observed. However, all the patients in this study where radiotherapy was the main treatment eventually died of their disease [4].

### Chemotherapy

The role of chemotherapy as an adjuvant to surgery is not yet established. There are no evidence-based guidelines regarding neoadjuvant or adjuvant chemotherapy for the treatment of UC [2]. Shao et al. [3] described a case in which recurrence free survival of 15 years is reached in a patient without metastatic disease by performing an extended partial cystectomy followed by an adjuvant chemotherapy-5-FU-based regimen. Multimodality therapy is an area that should be further explored for its potential to reach better outcomes.

UC is often diagnosed very late due to its clinically silent character. Systemic chemotherapy has the potential to be life-prolonging in this group, although the 5-year overall survival is less than 20%. There is clearly a need for more effective systemic treatment regimens [1, 3].

The two regimens with the highest response rates are 5-FU and cisplatin-based combination therapies. This can be explained by the histological and clinical similarities between UC and colon adenocarcinomas, on one hand, and urothelial carcinomas of the bladder, on the other. 5-FU-based chemotherapy was found overall more effective than cisplatin-based regimes [1]. However, the combination of both 5-FU and cisplatin could provide the strongest antitumor effect and thus the highest benefit for UC patients [1, 3, 5].

### Targeted Therapy

To date, little is known about the molecular background of UC. A large analysis was recently performed by Reis et al. [5], where case studies were collected to evaluate the potential pathogenic and therapy-relevant mutations, alterations in DNA mismatch repair (MMR), proteins/microsatellite instability (MSI), and programmed cell death ligand 1 (PD-L1) expression.

It could be concluded that on a molecular level the urachal adenocarcinomas are a distinct entity, demonstrating close similarities to the colorectal adenocarcinomas. As in colorectal cancer, therapy with epidermal growth factor receptor (EGFR)-inhibitors shows promising results. Furthermore, alterations in PD-L1 status, intracellular signal transduction pathways (RAS/RAF/PI3K), ERBB2 (HER2), MET, FGFR1, and PDGFRA have been found. Further research into immunotherapy and targeted therapeutic strategies is necessary [1, 3, 5].

## Discussion

The UC is a very rare and aggressive tumor that often presents in an advanced stage of disease. In that context, large prospective randomized clinical studies evaluating treatment success for different therapies are very difficult to conduct [20] To date, there are no evidence-based guidelines for the treatment of UC.

As described above, a clear distinction needs to be made between treatment for limited local disease and extensive systemic disease. Furthermore, an important distinction must be made within the group of extensive disease between patients with distant metastases (to the lungs, liver, bone, …) and patients with isolated PC. Patients with PC do not have the same benefit from systemic chemotherapy as patients with distant metastases. This can be explained by a physiological barrier formed by the peritoneum, also known as the peritoneal-plasma barrier [35]. This pharmacological barrier, comparable to the blood–brain barrier, makes it harder for drugs to pass in or out of the peritoneal cavity. This has an important impact on the pharmacokinetics of chemotherapy administered systemically. A systemically administered chemotherapeutic agent will quickly spread through the bloodstream to the various organs. The relatively low blood supply of the peritoneal membrane ensures that only a small amount of the drug will reach the peritoneal space. In contrast, chemotherapy administered straight into the peritoneal cavity ensures for a high concentration gradient without high resorption into the bloodstream. This method provides high locoregional cytotoxicity with minimal systemic toxicity [35].

CRS in combination with HIPEC has already proven to be effective in multiple abdominal cancers and is currently the gold standard for the treatment of isolated peritoneal metastatic colorectal cancer, appendiceal cancer, pseudomyxoma peritonei, and peritoneal mesothelioma [36]. Furthermore, promising results are achieved in therapy for peritoneal dissemination from gastric cancer and ovarian cancer when compared to palliative treatment. Over time, CRS and HIPEC has become safer and provided better outcomes [36]. When performed at a high-volume center, this procedure can achieve long-lasting patient survival and even cure some of the patients [28, 36, 37].

Some publications have shown similar positive results in the treatment of PC of UCs by means of CRS and HIPEC. Table 3 gives an overview of the publications and their results of the last 25 years. Most reports contain a single case or a small number of patients. The current two cases included there are only 70 cases in total known in the English literature in which patients with PC of UC underwent CRS and HIPEC. The largest series of patients was reported on by Mercier et al. [28] in 2018, followed by Mertens et al. [25] in 2019 and Liu et al. [27] in 2015 [25]–[33].

**Table 3:** Overview of cases in which patients received CRS/HIPEC as treatment for peritoneal dissemination originating from UC

Author	Loggie et al. [33]	De Bree et al. [29]	Sugarbaker et al. [30]	Martinez et al. [31]	Krane et al. [26]	Honoré et al. [32]	Liu et al. [27]	Mercier et al. [28]	Mertens et al. [25]	Current cases
Year	1997	2000	2008	2010	2012	2015	2015	2018	2019	2022
Number (n) of patients	1	1	2	1	5	3	9	36	10	2
Gender male *n* (%)	1 (100)	1 (100)	1 (50)	1 (100)	5 (100)	NA	4 (44)	24 (66.7)	9 (90)	1 (50)
Mean age in years (SD) Median age in years (range)	35 NA	34 NA	39.5 39.5 (32–47)	32 NA	NA 36 (18–59)	NA NA	50.9 48 (27–65)	NA 42.7 (20.6–61.1)	49.4 (15.3) NA	45.5 45.5 (42–49)
Histological type (%)	Well-differentiated signet-cell Aca (100)	Mucinous Aca (100)	Mucinous Aca (100)	Mucinous cystoAca (100)	NA	Mucinous (100)	Mucinous Aca (100) *4 signet ring cell (44)*	NA	Mucinous (100) *1 signet ring cell* *2 mixed type*	Mixed mucinous and enteric (2)
Staging	NA	NA	NA	NA	NA	NA	NA	NA	Sheldon III/IV	Sheldon III
Surgical intervention	Initial excision of the umbilicus and midline fascia CRS/HIPEC (1)	Initial microscopically radical excision CRS/HIPEC (1)	Initial radical excision and lavage (1) CRS/HIPEC (4)	CRS/HIPEC	Urachal excision (3) Cystoprostatectomy with ileal conduit (1) CRS/HIPEC (6)	CRS/HIPEC (3)	Prior local surgery (8) CRS/HIPEC (9)	CRS/HIPEC (36)	CRS/HIPEC (11)	CRS/HIPEC (2)
Complete resection status at time of CRS (%)	0 (0)	NA	2 (100)	1 (100)	2 (40)	NA	7 (77.8)	24 (66.7)	7 (70)	2 (100)
Chemotherapy (*n*) *Neoadjuvant* *HIPEC* *Adjuvant*	+ (1) + (1) + (1)	- + (1) + (1)	- + (4) + (2)	- + (1) -	+ (4) + (6) + (3)	NA + (3) NA	- + (9) -	+ (17) + (36) + (10)	- + (11) +	- + (2) -
Radiation therapy	Palliative for bony metastases (1)	Local external radiotherapy (1)	-	-	Concurrent postoperative (1)	NA	-	-	Brachytherapy (10)	-
Median follow-up in months (range)	31	NA	75 (18–132)	24	27 (21–87)	20 (14–37)	NA	48 (34.9–69.4)	96.8 (38.2–121.5)	102 (60–144)
Median survival in months (range)	31	NA	75 (18–132)	24	27 (21–87)	20 (14–37)	27.5 (6–61)	58.5 (11.2–105.9)	NA	102 (60–144)
Disease specific survival *2 years* *3 years* *5 years*	100% 0% 0%	NA NA NA	50% 50% 50%	100% NA NA	60% 20% 20%	NA NA NA	77.8% 55.6% 22.2%	NA 55.4% 46.2%	66.7% NA 55.6%	100% 100% 100%
Recurrence n (%)	1 (100)	NA	1 (50)	0 (0)	5 (100)	1 (33)	1 (11)	NA	5 (50)	0 (0)
Death by disease n (%)	1 (100)	NA	1 (50)	0 (0)	5 (100)	1 (33)	0 (0)	NA	4 (40)	0 (0)
*Major complications after CRS/HIPEC*	NA	NA	NA	NA	40%	NA	0%	37.9%	20%	50%

*NA* data not available, *Aca* adenocarcinoma.

+ applied.

- not applied.

It is stated that patients with UC have an overall 5-year survival rate of less than 50%. This is mainly because the diagnosis is often made at an advanced stage of disease, with 15–20% of patients presenting with distant metastases. Palliative chemotherapy or radiation therapy used to be the only treatment option for this group, leaving the patients with a dismal prognosis. Median survival of 1.3 years after starting therapy is established [1, 25, 27]. Considering the data presented in the publications mentioned in Table 4, especially from the last decade, CRS with HIPEC provided a clear survival benefit with even curation in some patients with isolated PC. The two new cases presented in this manuscript, for example, showed no signs of recurrence following respectively 5 and 12 years after treatment. When performed at high volume centers, the morbidity rates become acceptable as well [25]–[33].

**Table 4:** Chemotherapy used in HIPEC

Type	*n*	Study (*n* = times used)
Mitomycin C	32	(1) Loggie et al. [33] (1) De Bree et al. [29] (1)* Sugarbaker et al. [30] (5)^1^ Krane et al. [26] (15) Mercier et al. [28] (10) Mertens et al. [25]
Oxaliplatin	5	(1) Martinez et al. [31] (1)^1^ Krane et al. [26] (3) Mercier et al. [28]
Cisplatin	2	(1)* Sugarbaker et al. [30] (1) Mercier et al. [28]
Mephalan	2	(2) Mercier et al. [28]
Mitomycin C + cisplatin	17	(9) Liu et al. [27] (8) Mercier et al. [28]
Oxaliplatin + irinotecan	4	(4) Mercier et al. [28]
Cisplatin + doxorubicin	3	(1)* Sugarbaker et al. [30] (2) Mercier et al. [28]
Mitomycin C + doxorubicin	3	(1) Sugarbaker et al. [30] (2) Mercier et al. [28]
Missing data	4	(3) Honoré et al. [32] (1) Mertens et al.[25]

*The same patient was treated with CRS and HIPEC three times.

^1^The same patient was treated with CRS and HIPEC two times.

As described, there are several options regarding the specific therapy in this group of patients. Cases were published in which neo-adjuvant and/or adjuvant chemotherapy was used, whether or not in combination with radiation therapy (Table 3). There is, however, no standard chemotherapy regimen or radiation strategy available and their contribution to survival is not clear [3, 20, 25]–[30, 33].

In addition, a large variety in HIPEC regimens and time of exposure was seen in different centers. None of these treatment options have been or will be studied in large, randomized trials due to the scarcity of the condition. In the available data mitomycin C was used as monotherapy 32 times and in combination with cisplatin 17 times, making these two regimes the most preferred. Mercier et al. [28] already suggested these to have optimal efficiency. However, a formal recommendation cannot be made, since no statistical analysis can be done.

Several factors have been reported to be associated with prognosis. Achieving complete cytoreduction (CC-0 or CC-1) is an important independent factor influencing the patients’ prognosis. Therefore, it is advised that the patient is treated in a specialized center as soon as possible after diagnosis. Systematically performing a radical cystectomy has not proven to provide survival benefit, compared to partial cystectomy. Even though the presence of distant metastases and lymphatic invasion have shown to have a poor prognosis, no study has been able to proof a survival benefit for lymph node dissection [25, 28]. Other important factors influencing prognosis are the tumor size and histological subtype. Especially presence of signet ring cell differentiation is described in multiple studies to have a poor prognosis, whereas the mucinous tumor type does not have a prognostic effect on survival [1, 5, 27].

Although remarkable results have been achieved using CRS/HIPEC, close follow-up is still recommended in these patients due to the high malignancy of the tumor and the possibility of recurrence. MRI and tumor markers can be used for this purpose [17, 27].

## Conclusion

UC is a very rare and aggressive malignancy for which there is currently no standardized treatment. Several treatment options are known. However, none of these have ever been or will ever be tested in large, randomized trials due to the scarcity of the tumor. This manuscript discusses the importance of considering PC of UC origin as a separate entity, instead of as part of distant metastatic disease. It is demonstrated that CRS/HIPEC can provide satisfactory long-term oncological outcomes with acceptable morbidity and mortality rates. Our conclusion is therefore to consider CRS/HIPEC as a safe and feasible treatment option that may be curative in at least some patients. To obtain more results regarding this treatment and its outcome, all patients should be prospectively entered in a worldwide registry collecting all data.

## References

[1] Szarvas T *et al* (2016) Clinical, prognostic, and therapeutic aspects of urachal carcinoma—a comprehensive review with meta-analysis of 1,010 cases. Urol Oncol Semin Original Inv 34(9) Elsevier Inc. 388–398. 10.1016/j.urolonc.2016.04.012 10.1016/j.urolonc.2016.04.012 27267737

[2] van Allen J (2021). A rare case of urachal adenocarcinoma with bone marrow metastasis. BMJ Case Rep.

[3] Shao G (2022). Clinical, pathological, and prognostic analysis of urachal carcinoma. Urol Int.

[4] Sheldon CA, Clayman Rv, Gonzalez R, Williams RD and Fraley EE (1984) Malignant urachal lesions. J Urol 131(1):1–8. 10.1016/s0022-5347(17)50167-6 10.1016/s0022-5347(17)50167-6 6361280

[5] Reis H, Szarvas T (2019). Urachal cancer—current concepts of a rare cancer. Pathologe.

[6] Sharma P, Eigbire G, Sharma R (2021). Small bowel obstruction due to metastatic urachal adenocarcinoma: a rare presentation. Cureus.

[7] Sugarbaker PH (1999). Successful management of microscopic residual disease in large bowel cancer. Cancer Chemother Pharmacol.

[8] Paul BK, Ihemelandu C, Sugarbaker PH (2018). Prior surgical score: an analysis of the prognostic significance of an initial nondefinitive surgical intervention in patients with peritoneal carcinomatosis of a colorectal origin undergoing cytoreductive surgery and perioperative intraperitoneal chemotherapy. Dis Colon Rectum.

[9] Kilickap S, Akgul E, Aksoy S, Aytemir K, Barista I (2005). Doxorubicin-induced second degree and complete atrioventricular block. Europace.

[10] Chen X, Kang C and Zhang M (2019) Imaging features of urachal cancer: a case report. Front Oncol 9. 10.3389/fonc.2019.01274 10.3389/fonc.2019.01274 PMC6901919 31850195

[11] Gopalan A (2009). Urachal carcinoma: a clinicopathologic analysis of 24 cases with outcome correlation. Am J Surg Pathol.

[12] Chen Z-F *et al *(2008) [Clinical analysis of 14 cases of urachal carcinoma]. Ai Zheng 27(9):966–9.[Online]. Available: http://www.ncbi.nlm.nih.gov/pubmed/18799037 18799037

[13] Yu YD *et al *(2021) The prognosis and oncological predictor of urachal carcinoma of the bladder: a large scale multicenter cohort study analyzed 203 patients with long term follow-up. Front Oncol. 10.3389/fonc.2021.683190 10.3389/fonc.2021.683190 PMC8202399 34136407

[14] Koster IM, Cleyndert P, Giard RWM (2009). Urachal carcinoma. Radiographics.

[15] Yu J-S, Kim KW, Lee H-J, Lee Y-J, Yoon C-S, Kim M-J (2001). Urachal remnant diseases: spectrum of CT and US findings. Radiographics.

[16] Stokkel LE (2020). Diagnostic laparoscopy and abdominal cytology reliably detect peritoneal metastases in patients with urachal adenocarcinoma. Ann Surg Oncol.

[17] Siefker-Radtke AO *et al *(2003) Multimodality management of urachal carcinoma: the M. D. Anderson Cancer Center experience. J Urol 169(4):1295–1298. 10.1097/01.ju.0000054646.49381.01 10.1097/01.ju.0000054646.49381.01 12629346

[18] Bao B, Hatem M, Wong JK (2017). Urachal adenocarcinoma: a rare case report. Radiol Case Rep.

[19] Henly DR, Farrow GM, Zincke H (1993). Urachal cancer: role of conservative surgery. Urology.

[20] Ashley RA (2006). Urachal carcinoma: clinicopathologic features and long-term outcomes of an aggressive malignancy. Cancer.

[21] Herr HW, Bochner BH, Sharp D, Dalbagni G, Reuter VE (2007). Urachal carcinoma: contemporary surgical outcomes. J Urol.

[22] Molina JR, Quevedo JF, Furth AF, Richardson RL, Zincke H, Burch PA (2007). Predictors of survival from urachal cancer. Cancer.

[23] G. P. Paner, A. Lopez-Beltran, D. Sirohi, and M. B. Amin, Updates in the pathologic diagnosis and classification of epithelial neoplasms of urachal origin, 2016. [Online]. Available: http://www.anatomic 10.1097/PAP.0000000000000110 26849813

[24] Spiess PE, Correa JJ (2009). Robotic assisted laparoscopic partial cystectomy and urachal resection for urachal adenocarcinoma. Int Braz J Urol.

[25] Mertens LS (2019). Long-term survival after cytoreductive surgery and hyperthermic intraperitoneal chemotherapy (HIPEC) for patients with peritoneal metastases of urachal cancer. Eur J Surg Oncol.

[26] L. S. Krane, A. K. Kader, and E. A. Levine, Cytoreductive surgery with hyperthermic intraperitoneal chemotherapy for patients with peritoneal carcinomatosis secondary to urachal adenocarcinoma, J Surg Oncol, vol. 105, no. 3, pp. 258–260, 2012, 10.1002/jso.22081 10.1002/jso.22081 22271499

[27] Liu Y (2015). Cytoreductive surgery plus hyperthermic intraperitoneal chemotherapy for Pseudomyxoma peritonei arising from urachus. Ann Surg Oncol.

[28] Mercier F (2018). Peritoneal carcinomatosis of urachus origin treated by cytoreductive surgery and hyperthermic intraperitoneal chemotherapy (HIPEC): an international registry of 36 patients. Ann Surg Oncol.

[29] de Bree E, Witkamp A, van de Vijver M and Zoetmulde F (2000) Unusual origins of Pseudomyxoma peritonei 10.1002/1096-9098(200012)75:4<270::aid-jso9>3.0.co;2-v 11135270

[30] P. H. Sugar-baker, P. H. Sugarbaker, M. Verghese, T. D. Yan, and E. Brun, Management of mucinous urachal neoplasm presenting as pseudomyxoma peritonei, 2008. 10.1177/030089160809400515 19112949

[31] Martínez A, Ferron G, Mery E, Gladieff L, Delord JP, Querleu D (2012). Peritoneal pseudomyxoma arising from the urachus. Surg Oncol.

[32] Honoré C, Goéré D, Macovei R, Colace L, Benhaim L, Elias D (2016). Peritoneal carcinomatosis from unusual cancer origins: is there a role for hyperthermic intraperitoneal chemotherapy?. J Visc Surg.

[33] Loggie BW, Fleming RA, Hosseinian AA (1997). Peritoneal carcinomatosis with urachal signet-cell adenocarcinoma. Urology.

[34] Alzahrani N, Valle SJ, Liauw W and Morris DL (2017) Unusual indications of cytoreductive surgery and hyperthermic intraperitoneal chemotherapy: a review of outcomes and report of the literature, in *Unusual Cases in Peritoneal Surface Malignancies*, Springer International Publishing. 47–60. 10.1007/978-3-319-51523-6_4

[35] Jacquet P and Sugarbaker PH (1996) Peritoneal-plasma barrier. 53–63. 10.1007/978-1-4613-1247-5_4 10.1007/978-1-4613-1247-5_4 8849943

[36] Passot G (2016). What made hyperthermic intraperitoneal chemotherapy an effective curative treatment for peritoneal surface malignancy: a 25-year experience with 1,125 procedures. J Surg Oncol.

[37] Mielko J, Rawicz-Pruszyński K, Sędłak K, Gęca K, Kwietniewska M, Polkowski WP (2020). Cytoreductive surgery and hyperthermic intraperitoneal chemotherapy for peritoneal surface malignancies: learning curve based on surgical and oncological outcomes. Cancers (Basel).

